# Isolating the impact of tissue heterogeneities in high dose rate brachytherapy treatment of the breast^☆^

**DOI:** 10.1016/j.phro.2025.100737

**Published:** 2025-02-22

**Authors:** Jules Faucher, Vincent Turgeon, Boris Bahoric, Shirin A. Enger, Peter G.F. Watson

**Affiliations:** a Medical Physics Unit, McGill University, Cedars Cancer Centre, 1001 boul Décarie, Montréal, QC H4A 3J1, Canada; b Department of Radiation Oncology, CISSS de la Montérégie-Centre, 3120 boul Taschereau, Greenfield Park, QC J4V 2H, Canada; c Department of Radiation Oncology, Jewish General Hospital, 3755 Ch De La Cote-Sainte-Catherine, Montreal, QC H3T1E2, Canada; d Lady Davis Institute for Medical Research, Jewish General Hospital, 3755 Ch De La Cote-Sainte-Catherine, Montreal, QC H3T1E2, Canada

**Keywords:** HDR brachytherapy, Heterogeneity corrections, Breast cancer, Monte Carlo method

## Abstract

**Background and purpose:**

Clinical brachytherapy treatment planning is performed assuming the patient is composed entirely of water and infinite in size. In this work, the effects of this assumption on calculated dose were investigated by comparing dose to water in water (D_w,w_) in an unbound phantom mimicking TG-43 conditions, and dose to medium in medium (D_m,m_) for breast cancer patients treated with high dose rate brachytherapy.

**Materials and methods:**

Treatment plans for 123 breast cancer patients were recalculated with a Monte Carlo-based treatment planning software. The dwell times and dwell positions were imported from the clinical treatment planning system. The dose was computed and reported as D_w,w_ and D_m,m_. Dose-volume histogram (DVH) metrics were evaluated for target volumes and organs at risk.

**Results:**

D_w,w_ overestimated the dose for most studied DVH metrics. The largest median overestimations between D_m,m_ and D_w,w_ were seen for the planning target volume (PTV) V_200%_ (5.8%), lung D_0.1 cm_^3^ (6.0%) and skin D_0.1 cm_^3^ (4.2%). The differences between D_m,m_ and D_w,w_ were statistically significant for all investigated DVH metrics_._ The PTV V_90%_ had the smallest deviation (0.7%).

**Conclusion:**

There was a significant difference in the DVH metrics studied when tissue heterogeneities and patient-specific scattering are accounted for in high dose rate breast brachytherapy. However, for the studied patient cohort, the clinical coverage goal (PTV V_90%_), had the smallest deviation.

## 1 Introduction

Breast cancer, which has the highest occurrence amongst women [1], is often treated using radiation therapy post-conservative surgery [2]. Radiation is delivered to the entire ipsilateral breast as whole breast irradiation (WBI) or only to the vicinity of the surgical bed as accelerated partial breast irradiation (APBI) [3]. Several studies have reported that for stage 0 to 2 breast cancers, APBI offers the same local control and recurrence rates as WBI, with good to excellent cosmetic results [4], [5], [6], [7], [8], [9]. In APBI delivered with high dose rate (HDR) brachytherapy, 34 Gy in 10 fractions is prescribed to the tumor bed post-lumpectomy [5]. Depending on the size of the breast and the extent of the lesion, 10 to 25 interstitial catheters are inserted in the patient's breast to adequately cover the surgical cavity [10].

Traditionally, clinical treatment planning systems (TPSs) calculate absorbed dose according to the formalism established by the American Association for Physicists in Medicine (AAPM) in their Task Group report 43 (TG-43) [11]. This formalism assumes that the radiation source is surrounded by an infinite and homogeneous water medium. Dose heterogeneities introduced by the composition of different tissue types, applicator/source material, mass densities or patient finite dimensions are ignored. This approximation allows the use of an analytical solution which is computationally inexpensive. As such, treatments can be planned and optimized in a couple of minutes. Limitations caused by the approximations of TG-43-based dose calculations have been discussed in several studies, of which we refer to a few examples [12], [13], [14], [15], [16]. For brachytherapy treatment of breast cancer, clinical TPSs based on the TG-43 dose formalism cannot accurately calculate the absorbed dose to the tumor volume and surrounding organs at risk (OARs) due to lack of full scatter conditions, since the radiation source is placed close to the tissue-air interface [17].

In 2012, AAPM report TG-186 was published, guiding the use of model-based dose calculation algorithms (MBDCAs) [18]. MBDCAs require voxel-by-voxel tissue and applicator elemental composition and mass density assignments. Since dose calculations are performed on segmented patient computed tomography (CT) images, the finite dimension of the patient is implicitly considered. However, to date, the treatment planning is still performed according to TG-43. The recommendation of transitioning from TG-43 to TG-186-based dose calculations by professional medical physics and radiation oncology societies will require data that correlates clinical outcomes to the computed doses of MBDCAs.

In previous work, Peppa *et al.* investigated dose differences between TG-43 and Monte Carlo (MC) based dose calculations for 57 ^192^Ir HDR breast brachytherapy patients [19]. The authors concluded that although TG-43-based dose calculations show a statistically significant overestimation of most dose-volume histogram (DVH) indices used for plan evaluation, differences are not clinically significant. However, the authors also point out that improved dose computations based on MBDCAs could be important for re-irradiation purposes, inter-comparison, and/or the assessment of secondary cancer induction risk, where accurate dose calculations in the whole patient anatomy are necessary.

However, this study and similar work uses different algorithms for volume or dose calculations [20], [21], [22], [23], [24], potentially introducing systematic errors. In infinite water phantoms, there can be slight disagreements of dose distributions computed with the analytical formalism used in clinical TPSs and with MC methods [25]. Additionally, the volume of structures, and subsequently DVHs, are calculated differently between TPSs [26]. To directly evaluate the impact of the TG-43 assumptions, the same TPS and MC dose calculation algorithm should be used to compute both the dose in a model of the tissues of a patient and an unbound water phantom.

In this study, the effects of tissue heterogeneities and finite patient dimensions on DVH metrics for breast HDR brachytherapy treatments were studied by calculating dose to medium in medium (D_m,m_) and dose to water in water (D_w,w_) in a patient cohort of 123 patients. To isolate the effects of the infinite water patient assumption of TG-43, the dose in water and the dose in the medium were computed using the same MC-based TPS.

## 2 Materials and methods

### 2.1 Patient cohort

Dose calculations were performed for a cohort of 123 breast cancer patients treated with post-surgery HDR brachytherapy at the Jewish General Hospital (Montreal, QC, Canada). This cohort comprises all patients who received this treatment at the moment this study received approval from our institutional review board (2020). The average patient age at the time of brachytherapy was 63 years. These patients were selected following the ASTRO guidelines for APBI [27].

### 2.2 Treatment planning

The standard prescription dose was 34 Gy in 10 fractions. Patient CT images were acquired after the insertion of catheters. Clinical target volume (CTV) and planning target volume (PTV) were contoured on this image set. Our departmental definition of CTV, PTV and planned target volume for evaluation (PTV_Eval) follow the recommendations by the National Surgical Adjuvant Breast and Bowel Project (NSABP)/Radiation Therapy Oncology Group (RTOG) protocol B-39/0413 [3]. In the NSABP/RTOG protocol B-39/0413, the PTV corresponds to a 15 mm uniform expansion of the excision cavity, and the PTV_EVAL, which is generated to allow evaluation of the dose delivered to the idealized target volume, is defined as the PTV bounded by 5 mm within the skin and the breast pectoralis interface. The chest wall is excluded from the PTV. In this study, for each patient, post-implant dose calculations were performed on the set of CT images in the DICOM format mentioned above. Contours were imported from DICOM RT Structure Set files. Dwell positions were extracted from clinical DICOM RT Plan files.

### 2.3 Tissue assignment schemes

For each image set, the patient geometry was segmented in 1 mm^3^ voxels. Patient tissue and mass density were assigned voxel by voxel according to two tissue assignment schemes. In the first scheme, tissue assignment was based on TG-43 recommendations, where patient tissues and the surrounding air were assigned the elemental composition of water and unit density such that D_w,w_ was calculated. It is important to note that this method of assigning all CT voxels as water is not equivalent to TG-43 since we do not use analytical formalisms to compute the dose. This ensures that volumes are computed similarly across calculation methods. In our TPS, a volume is calculated by summing up the volumes of voxels whose centers are contained within a contour outline. Meanwhile, Oncentra uses a distance map to create a triangulated surface (Oncentra Brachy Physics and Algorithms User Manual). Volume calculation algorithms and their impact on DVHs are discussed in more detail in Kirisits *et al.* [28].

In the second scheme, tissue composition and mass densities were derived from the CT scanner's Hounsfield unit to density calibration curve (Supplementary Table S1) to create patient models from which D_m,m_ was calculated. The density of tissue in a given class is interpolated from CT numbers and their elemental compositions were assigned based on recommendations from ICRU’s report 46 [29] (Supplementary Table S2).

Report TG-186 recommends assigning different compositions to mammary glands and adipose tissue, or assigning a homogeneous mixture of 80 % adipose and 20 % glands for the whole breast only in cases where glands and adipose cannot be differentiated in images of poor quality [18]. Some of the images used in this work lacked the quality to accurately segment glands from fatty tissue. However, areas of higher gland density were recognizable from adipose. As a compromise between the recommendations of Report TG-186, breasts were segmented by assigning the composition of adipose to fatty tissue, and a homogeneous mixture of 50 % adipose and 50 % glands to areas of high glandularity.

### 2.4 Monte Carlo simulations

Calculations were performed by using a validated treatment planning software, RapidBrachyMCTPS [25], with an MC dose calculation engine based on Geant4 [30], [31]. An explicit simulation of radioactive decay using photon decay spectra from the Evaluated Nuclear Structure Data File (ENSDF) [32] was used to generate 10^8^ decay histories. Cross-section libraries EPDL97 and EADL97 were used for photon and atomic data, respectively [33], [34]. The PENELOPE low-energy electromagnetic physics list was used with the default transport parameters. To improve computational efficiency, the dose was approximated by collisional kerma, due to the short range of secondary electrons compared to the voxel size [11] and was scored with a track length estimator. On average, the Type A uncertainty was 0.64 % at the 100 % isodose line. A summary of the MC methods used is presented in Supplementary Table S3 as suggested by AAPM TG-268 [35]. The MC calculations were performed on the Digital Research Alliance of Canada’s Cedar cluster.

### 2.5 Dose/volume indices

In this study, cumulative DVH parameters were evaluated using D_m,m_ and D_w,w_ to investigate the effect of tissue heterogeneities on the dose calculation for breast HDR brachytherapy treatments. Based on the NSABP/RTOG protocol B-39/0413, the dose homogeneity index (DHI), defined as 1 − V_150_/V_100_, for the entire body and the PTV V_90%_ were evaluated [3]. In addition, the D_100%_ of the CTV, the V_200%_, V_100%_, and D_90%_ of the PTV to provide further insight on the homogeneity and coverage of the target. The metrics V_x_, D_x_ and D_x cm_^3^ are defined in AAPM TG-263 [36].

The only dose limit to healthy tissue the NSABP/RTOG protocol recommends for APBI is that the maximum dose to the skin should not exceed the prescription. As such, our institution decided that the maximum doses to the lungs and chest wall should also not exceed the prescription in treatment plans. To understand how these dose limits are affected by heterogeneities, the D_0.1 cm_^3^ for the ipsilateral lung, chest wall, and skin were also evaluated. In this work, the D_0.1 cm_^3^ is used to estimate the max dose to OARs while being insensitive to random statistical fluctuations in MC simulations that could lead to maximum doses far greater than reality.

To compare the two dose calculation methods, DVH metrics were computed for each patient and p-values were computed using the Wilcoxon signed-rank test to establish the probability of creating one of the datasets by randomly sampling the other one. The Wilcoxon test was chosen because the data is paired (two-dose calculations per patient) and non-normally distributed, as confirmed by the visual inspection of histograms of D_m,m_ and D_w,w_.

## 3 Results

**Fig. 1:**
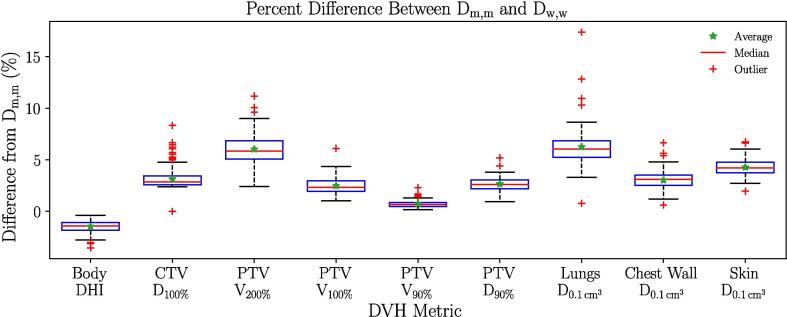
Box plot representing the percent difference between D_m,m_ and D_w,w_. The horizontal line is the median, and the top and bottom edges are the 75th and 25th percentile. The whiskers extend to the outermost data point not considered an outlier, and the outliers are indicated with red crosses (+). (For interpretation of the references to colour in this figure legend, the reader is referred to the web version of this article.)

D_w,w_ overestimated D_m,m_ for every DVH metric studied, as shown in Table 1. Even when considering patients individually, DVH metrics obtained from D_w,w_ were higher than the ones computed from D_m,m_. Consequently, the Wilcoxon test yielded p-values below 0.05. The skewness in the difference of the data required for the Wilcoxon test to be valid is shown in Fig. 1. The non-normality of the data is shown in Supplementary Fig. S1. This figure also highlights how the spread of different PTV metrics increased going from D_w,w_ to D_m,m_.

**Table 1:** Median value and range (in %) of DVH metrics for both dose calculation methods.

**Structures and Indices**	**Median value and range(in %)of each DVH metrics**			
	**D_m,m_**		**D_w,w_**	
Body DHI	73.3	[56.2, 80.5]	72.1	[55.6, 79.8]
CTV D_100%_	105.9	[77.8, 123.5]	108.8	[81.5, 126.5]
PTV V_200%_	8.6	[5.9, 20.7]	9.1	[6.2, 21.9]
PTV V_100%_	91.7	[83.6, 94.3]	93.8	[87.3, 95.8]
PTV V_90%_	98.3	[92.9, 99.5]	98.9	[95.0, 99.9]
PTV D_90%_	101.7	[93.3, 106.2]	104.3	[97.7, 108.8]
Lungs D_0.1 cm_^3^	54.3	[14.7, 54.3]	57.0	[17.2, 85.6]
Chest Wall D_0.1 cm_^3^	88.2	[20.5, 163.8]	91.0	[20.6, 167.4]
Skin D_0.1 cm_^3^	91.9	[55.9, 200.8]	96.1	[58.0, 206.7]

The spread of differences between the DVH metrics obtained with D_m,m_ and D_w,w_ was greater for the lungs D_0.1 cm_^3^ and PTV V_200%_ as illustrated in Fig. 1. Both metrics also had the greatest median difference across all patients as shown in Table 2. Conversely, the PTV V_90%_ had both the smallest spread and median difference.

**Table 2:** Median and range of the difference (in %) between MC dose calculation methods. For each patient, the percent difference between dose calculations was computed. The median of these differences was then taken.

**Structures and Indices**	**Median differences (in %) between dose calculation methods**
Body DHI	−1.4 [-3.5, −0.4]
CTV D_100%_	2.9 [0.0, 8.3]
PTV V_200%_	5.8 [2.4, 11.2]
PTV V_100%_	2.3 [1.0, 6.1]
PTV V_90%_	0.7 [0.2, 2.3]
PTV D_90%_	2.6 [0.9, 5.2]
Lungs D_0.1 cm_^3^	6.0 [0.8, 17.4]
Chest Wall D_0.1 cm_^3^	3.1[0.6, 6.6]
Skin D_0.1 cm_^3^	4.2 [2.0, 6.7]

## 4 Discussion

Currently, the clinical dose calculation in interstitial breast HDR brachytherapy is based on D_w,w_ following the TG-43 formalism, where influences from heterogeneities and finite patient dimensions are ignored. This study showed that there are statistically significant differences in the calculated dose when tissue/applicator compositions, mass density, and patient-specific scatter conditions are introduced.

In this study, DVH metrics used to evaluate clinical coverage of the target volumes (CTV D_100%_, PTV V_100%_ and PTV V_90%_) were overestimated by D_w,w_ compared to their values calculated with D_m,m_. These results agreed with recent findings in the literature calculated for other tumor sites. Shoemaker *et al.* [20]reported a 6 % overestimation of CTV D_90%_ in rectum HDR brachytherapy with tungsten shielding when using D_w,w_-based dose calculations. Famulari *et al.* [21] investigated the effect of heterogeneity in prostate and oral tongue cancers treated with HDR brachytherapy. The authors reported an overestimation of PTV D_90%_ and V_100%_ of 1 % on average for both tumor sites treated with ^192^Ir when D_w,w_-based dose calculations were used.

In this study, the peak doses to three OARs (lung, chest wall, and skin) were investigated by evaluating D_0.1 cm_^3^. The differences between D_m,m_ and D_w,w_ were larger than the Type A uncertainty on the 100 % isodose line. This is particularly relevant for the skin and chest wall, for which the median D_0.1 cm_^3^ values were close to the prescription. The differences observed between D_m,m_ and D_w,w_ in lungs, skin and the chest wall agree with previous studies for breast HDR brachytherapy [19], [22], [23], [24]. Pantelis *et al.* used a mathematical phantom to compare the dose distributions between the TG-43 formalism and MC-based dose computations with finite phantom geometry [23]. For their single case, the authors found that TG-43 overestimates the D_0.1 cm_^3^ to the skin and to the lung by 4.7 % and 5.3 % respectively. However, Peppa *et al.* found that with TG-43, the maximum dose to the lung and the D_0.1 cm_^3^ to the skin were only overestimated by 2 % and 2.5 % respectively [19].

Our results suggest that the DVH metric with the smallest discrepancy between dose calculation formalisms is the PTV V_90%_. With a difference on par with the Type A uncertainty on the 100 % isodose line, we could not conclude that heterogeneities and finite patient dimensions significantly impacted the calculated dose. This makes sense because the tissues inside the PTV have mass energy absorption coefficients similar to water (Supplementary Fig. S2), and that 90 % of the volume of the PTV can potentially be far enough from tissue-air interfaces to be unaffected by the overestimation of scattered radiation close to skin. These results agreed with those of Poon *et al.* who found that TG-43 overestimates the dose to the PTV V_90%_ by 0.6 % [24]. These findings also align with Peppa *et al.*, who found a very small impact on PTV coverage when using the TG-43 formalism [19]. This indicates that the PTV V_90%_ is robust amongst dose calculation formalisms, and that its use to evaluate coverage, as per the NSABP/RTOG protocol B-39/0413, is a good way to minimize the impact of heterogeneities.

The p-values derived with the Wilcoxon test were extremely low across all DVH metrics. Mathematically, this statistical analysis tests whether the distribution of the differences between two paired groups is symmetric about zero so that two groups whose data distributions are skewed differently will yield a small p-value. Fig. 1 illustrates that the distributions of differences never spread across zero because the metrics of individual patients were always overestimated, which lead to the near-zero p-values observed.

A limitation of our study is that the accuracy of the dose to the PTV is limited by the presence of the high-density markers found on the catheters. In the simulation, high-density markers were identified as small boney features because of the simple CT-to-density curve used, and thus absorbed more dose than the surrounding soft tissue. While high-density markers are expected to absorb more dose than soft tissue, this dose is not imparted to the patient’s tissues. However, in the simulation, the dose to the markers were scored to the patient, thus increasing the volume of the PTV receiving high doses. To fix this systematic error, the markers should be identified on CT and be assigned the same Hounsfield units as their surrounding tissue. However, given that the markers represented less than 3 % of the volume of the PTV on average, the impact they had over the dose overestimation should have remained low.

Multiple studies have investigated the effects of heterogeneity and finite patient dimensions in HDR brachytherapy for breast cancer. Overall, the studies collectively reached the consensus that for this treatment, the TG-43 formalism leads to a notable overestimation of the dose in OARs compared to the absorbed dose calculated using MC simulations. These studies also found that the approximations of TG-43 had little impact on the DVH metric used as clinical coverage goal. These findings are reassuring since they imply that the errors induced by the TG-43 formalism are not detrimental to breast patient undergoing HDR interstitial brachytherapy. Our study, carried on a larger patient cohort than previous work, align with these findings.

## CRediT authorship contribution statement

**Jules Faucher:** Formal analysis, Investigation, Writing – original draft, Writing – review & editing, Visualization. **Vincent Turgeon:** Methodology, Data curation, Writing – original draft, Writing – review & editing. **Boris Bahoric:** Resources, Data curation, Writing – review & editing. **Shirin A. Enger:** Conceptualization, Methodology, Resources, Writing – review & editing, Supervision, Project administration. **Peter F. Watson:** Resources, Writing – review & editing, Supervision.

## Funding

This work was supported by the Cedars Cancer Foundation at the McGill University Health Centre.

## Declaration of competing interest

The authors declare that they have no known competing financial interests or personal relationships that could have appeared to influence the work reported in this paper.
