# Supplementary material for: Isolating the impact of tissue heterogeneities in high dose rate brachytherapy treatment of the breast
Source: Phys Imaging Radiat Oncol. 2025 Feb 22;33:100737. doi: 10.1016/j.phro.2025.100737 (PMC11910349; doi:10.1016/j.phro.2025.100737)
Supplement: Supplementary Data 1 [file mmc1.pdf]

Table S1: CT to density calibration curve. Densities are linearly interpolated from Hounsfield units.

Material	Density	Hounsfield Unit
Air	<0.001225	<-900
Lung	0.001225 - 0.260	-900 - -500
Adipose	0.260 - 0.970	-500 - -85
Breast 50/50	0.970 - 0.985	-85 - -70
Soft Tissue	0.985 - 1.020	-70 - -60
Heart	1.020 - 1.050	-60 - 200
Bone	1.050 - 1.920	200 - 500
Cortical Bone	>1.920	>500

Table S2: Composition and density of materials and tissues used for the simulations.

Material	Mass %					Mass Density (g/cm^3)
	H	C	N	O	Other Elements	
Air	0.00	0.012	75.527	23.178	Ar(1.283)	0.001225
Lung	10.30	10.50	3.10	74.90	Na(0.20), P(0.20), S(0.30), Cl(0.30), K(0.20)	0.260
Adipose	11.40	59.80	0.70	27.80	Na(0.10), S(0.10), Cl(0.10)	0.970
Breast 50/50	11.00	46.50	1.90	40.30	Na(0.10), P(0.10), S(0.20), Cl(0.10)	0.985
Soft Tissue	10.60	31.50	2.40	54.70	Na(0.10), P(0.20), S(0.20), Cl(0.10), K(0.20)	1.020
Heart	9.76	9.11	2.47	78.10	Na(2.47), Mg(0.019), P(0.10), K(0.20), Ca(0.023), Zn(0.008)	1.050
Bone	3.40	15.50	4.20	43.50	Na(0.10), Mg(0.20), P(10.30), S(0.30), Ca(22.50)	1.920
Cortical Bone	3.40	15.50	4.20	43.50	Na(0.10), Mg(0.20), P(10.30), S(0.30), Ca(22.50)	1.920
Steel	0.00	0.08	0.00	0.00	Si(1.00), Cr(19.00), Fe(67.92), Mn(2.00), Ni(10.00)	8.000
Plexiglass	8.00	60.00	0.00	32.00	N/A	1.190
Tungsten	0.00	0.00	0.00	0.00	W(100.00)	19.300
Silicone	8.20	32.40	0.00	21.60	Si(37.80)	1.140
Gold	0.00	0.00	0.00	0.00	Au(100.00)	19.320
Iridium	0.00	0.00	0.00	0.00	Ir(100.00)	22.560
PPSU	4.02	32.40	0.00	21.60	S(8.00)	1.290
Fiber Glass	2.58	27.33	0.22	36.58	B(2.04), Mg(3.66), Al(4.89), Si(16.66), Ca(6.04)	2.100

Table S3: Summary of Monte Carlo methods as per TG-286 recommendations.

**Property**	**Description**	**Reference**
Code, version	Geant4, RapidBrachyMCTPS	[25], [30], [31]
Validation	Previously validated.	[25]
Geometry	Voxelized geometry extracted from DICOM CT images and DICOM RT structure set files.	
Materials	Heterogeneous (TG-186), with elemental composition of tissues and CT-to-density conversion as presented in table A1 and A2.	[11], [13], [18], [29]
Source description	MicroSelectron v2. Explicit simulation of radioactive decay using photon decay spectra from ENSDF. Source positions and orientations imported from DICOM RT plan files.	[32]
Cross-sections	EPDL97, EADL97.	[33], [34]
Transport parameters	PENELOPE low-energy electromagnetic physics list with default transport parameters. Electron transport off. Production cut: 0.1 mm	
VRTs	Track length estimator using mass-energy absorption coefficient.	[13], [25]
Scored quantities	Absorbed dose (collisional kerma approximation) scored to water or medium.	
# histories/statistical uncertainty	10^8 radioactive decays per dwell position. Type A uncertainties ≤0.6% at the 100% isodose lines.	
Statistical methods	History-by-history method	
Postprocessing	Dose to voxels converted to DVH metrics using RapidBrachyMCTPS.	[25]

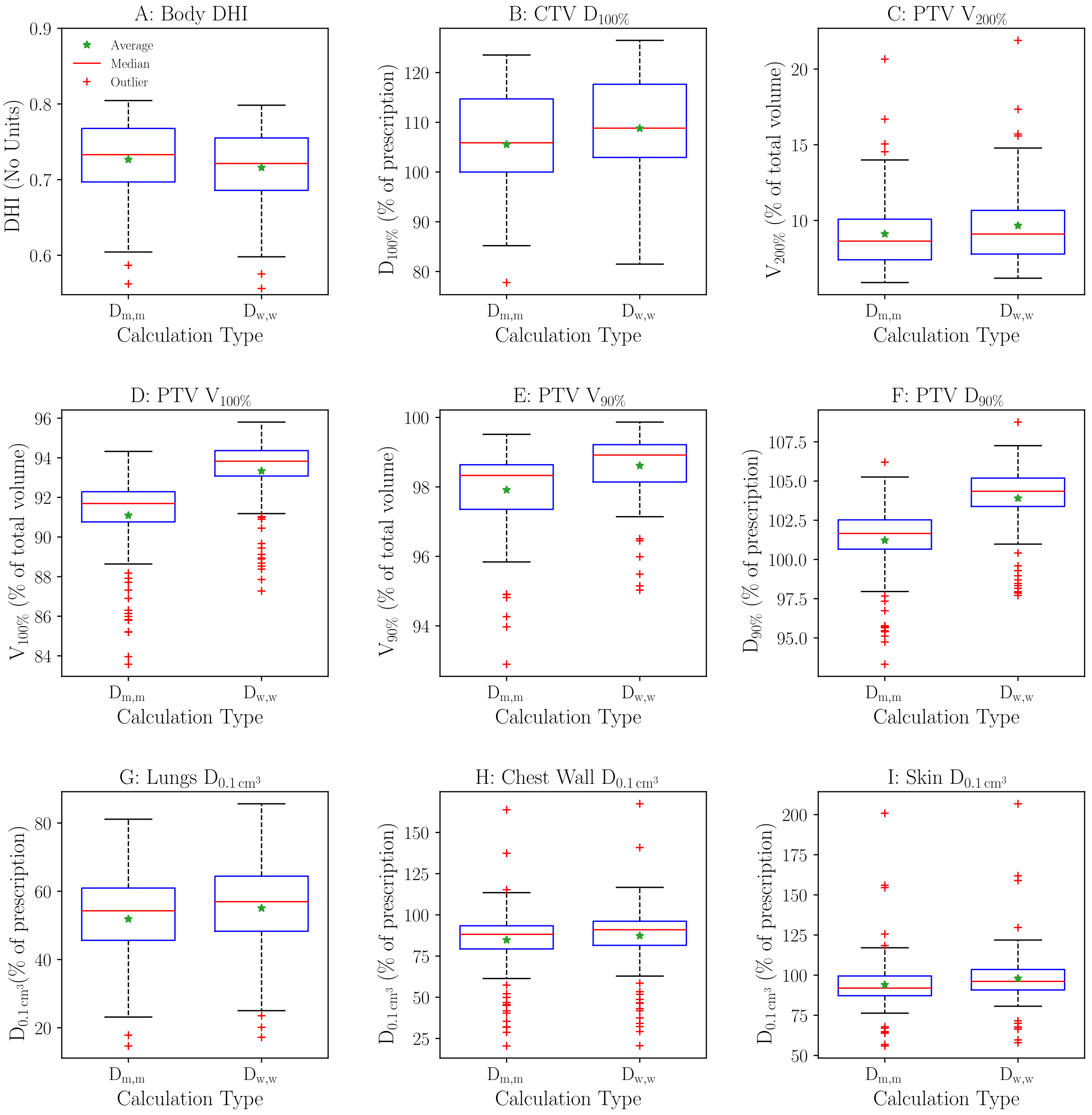

Figure S1: Box plot representing the distributions of DVH metrics for: A) DHI, B) CTV $D_{100\%}$, C) PTV $V_{200\%}$, D) PTV $V_{100\%}$, E) PTV $V_{90\%}$, F) PTV $D_{90\%}$, G) Lung $D_{0.1\,cm^3}$, H) Chest wall $D_{0.1\,cm^3}$ and I) Skin $D_{0.1\,cm^3}$. The horizontal line is the median, and the top and bottom edges are the 75th and 25th percentile. The whiskers extend to the outermost data point not considered an outlier, and the outliers are indicated with red crosses (+). All differences were statistically significant.

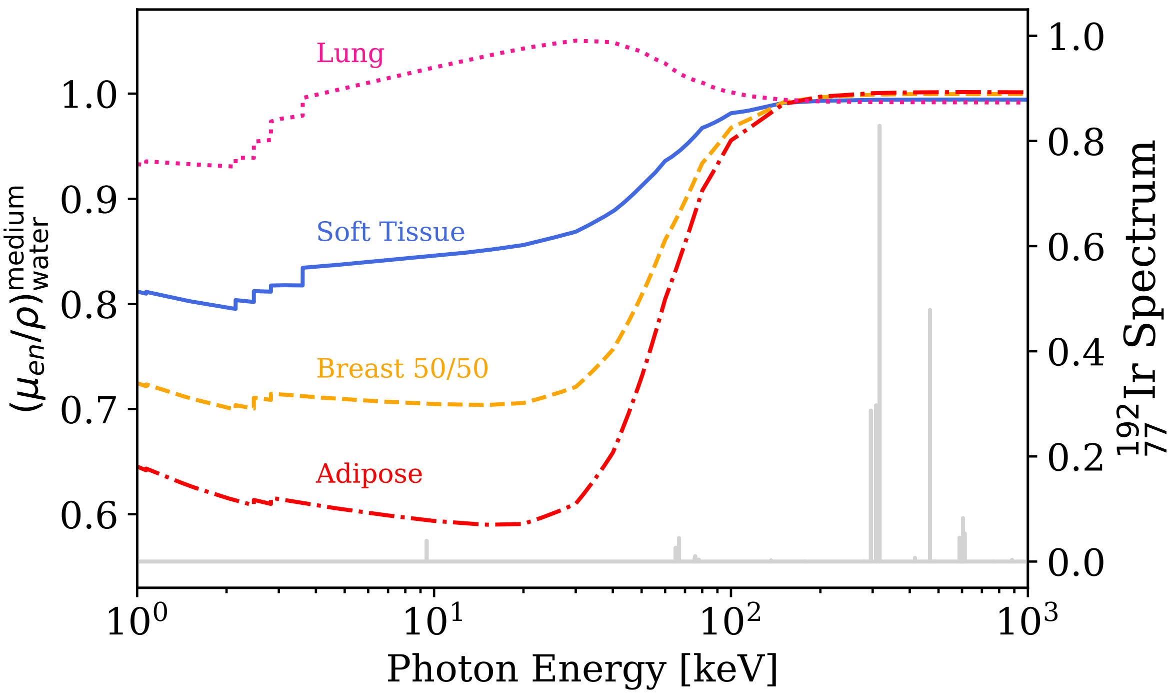

Figure S2: Ratios of mass energy absorption coefficients of different tissues to water. The photon spectrum of Iridium-192 was added in light gray to illustrate the range of energies of interest for this study. Characteristic X-rays produced by the source were also included (energies below 75 keV).
